# Computational study of structural changes in neuronal networks during growth: a model of dissociated neocortical cultures

**DOI:** 10.1186/1471-2202-12-S1-P203

**Published:** 2011-07-18

**Authors:** Jugoslava Aćimović, Tuomo Mäki-Marttunen, Marja-Leena Linne

**Affiliations:** 1 Department of Signal Processing, Tampere University of Technology, Tampere, Finland; 2 Department of Mathematics, Tampere University of Technology, Tampere, Finland

## 

Networks of neurons possess distinct structural organization that constraints generated activity patterns, and consequently, the functions of the system. The emergence of the network structure can be understood by studying the rules that govern growth of neurons and their self-organization into neuronal circuits. We analyze these rules using a computational model of growth developed for dissociated neocortical cultures. Compared to the growth *in vivo*, the cultures represent simplified two dimensional systems that still possess the intrinsic properties of single neurons although they lack the natural extracellular environment present *in vivo*. This setup provides a possibility to address in depth the selected mechanisms that affect neuronal growth. The collected structural data (through staining and microscopy) and electrophysiological data (using microelectrode arrays) facilitate validation of computational models. Neuronal growth in dissociated cultures has been examined in several studies in order to access the role of activity in network development [6],[7] or to extract the structural changes during growth from the recorded activity and identify the significant time points in network development [4]. In addition, two simulators of neuronal growth were recently published to aid the development of computational models [3],[9]. Their performance, in context of modeling neocortical cultures, is compared in [1].

The analyzed model consists of two types on neurons, most commonly observed in the neocortical cultures, the pyramidal cells and the nonpyramidal GABAergic cells, placed in a dish-like space with the density of cells corresponding to the experimental values. The phenomenological model that takes into account growth of every neurite is constructed using the description from the literature [3],[8]. It is compared to the model that defines only the overall shape of each neuritic field. We examine the critical time point in network development, i.e. the emergence of fully connected networks [2],[4], which is dependent on the overall growth speed of neurites. The local structural features are accessed using the frequency of motifs in networks [2],[5]. Local connectivity patterns, captured by the motif counts, depend on the shape of neurites and distribution of synaptic contacts along neurites. The goal of this study is to analyze model dynamics through evaluation of the proposed measures. The dependence on model parameters is examined in details, particularly, whether small variations in parameter values significantly affect both measures of network structure. The obtained conclusions are compared to the experimental findings from the literature [4,5].
